# High MICs for Vancomycin and Daptomycin and Complicated Catheter-Related Bloodstream Infections with Methicillin-Sensitive *Staphylococcus aureus*

**DOI:** 10.3201/eid2206.151709

**Published:** 2016-06

**Authors:** Rafael San-Juan, Esther Viedma, Fernando Chaves, Antonio Lalueza, Jesús Fortún, Elena Loza, Miquel Pujol, Carmen Ardanuy, Isabel Morales, Marina de Cueto, Elena Resino-Foz, Alejandra Morales-Cartagena, Alicia Rico, María P. Romero, María Ángeles Orellana, Francisco López-Medrano, Mario Fernández-Ruiz, José María Aguado

**Affiliations:** University Hospital–Research Institute 12 de Octubre, Madrid, Spain (R. San-Juan, E. Viedma, F. Chaves, A. Lalueza, E. Resino-Foz, A. Morales-Cartagena, M.Á. Orellana, F. López-Medrano, M. Fernández-Ruiz, J. María Aguado);; Hospital Universitario Ramón y Cajal, Madrid (J. Fortún, E. Loza);; University Hospital–Bellvitge Institute for Biomedical Research, Barcelona, Spain (M. Pujol, C. Ardanuy);; Hospital Universitario Virgen de la Macarena, Seville, Spain (I. Morales, M. de Cueto);; Hospital Universitario La Paz, Madrid (A. Rico, M.P. Romero)

**Keywords:** methicillin-sensitive Staphylococcus aureus, MSSA, bacteria, complicated bacteremia, vancomycin, daptomycin, MIC, septic thrombophlebitis, catheter-related bloodstream infection, CRBI

## Abstract

We investigated the prognostic role of high MICs for antistaphylococcal agents in patients with methicillin-sensitive *Staphylococcus aureus* catheter-related bloodstream infection (MSSA CRBSI). We prospectively reviewed 83 episodes from 5 centers in Spain during April 2011–June 2014 that had optimized clinical management and analyzed the relationship between E-test MICs for vancomycin, daptomycin, oxacillin, and linezolid and development of complicated bacteremia by using multivariate analysis. Complicated MSSA CRBSI occurred in 26 (31.3%) patients; MICs for vancomycin and daptomycin were higher in these patients (optimal cutoff values for predictive accuracy = 1.5 μg/mL and 0.5 μg/mL). High MICs for vancomycin (hazard ratio 2.4, 95% CI 1.2–5.5) and daptomycin (hazard ratio 2.4, 95% CI 1.1–5.9) were independent risk factors for development of complicated MSSA CRBSI. Our data suggest that patients with MSSA CRBSI caused by strains that have high MICs for vancomycin or daptomycin are at increased risk for complications.

## Abstract 2

Patients infected with these bacteria were more likely to have local endovascular complications.

*Staphylococcus aureus* bacteremia is an issue of concern because of high rates of illness and death for this condition (*1*). Some studies have identified high MICs for vancomycin (defined as an MIC >1.5 μg/mL by E-test) as a simple laboratory-based marker, which has been shown to be associated with a worse prognosis in methicillin-resistant *S. aureus* (MRSA) bacteremia in terms of treatment failure (*2*–*4*) and increased mortality rates (*5*,*6*). Such increased mortality rates associated with a high MIC for vancomycin has also been reported in methicillin-sensitive *S. aureus* (MSSA) bacteremia (*7**,**8*). However, the use of death as a study outcome and the fact that these studies were performed with patients with different sources of bacteremia make it difficult to interpret study results. Such findings could not be confirmed in large cohorts of patients with MRSA (*9*–*11*) or MSSA (*12*) bacteremia from different sources.

To avoid these confounding factors, we focused on a more homogeneous population of patients with MSSA catheter-related bloodstream infection (MSSA CRBSI) for whom specific efforts were devoted to optimize clinical management. In a previous retrospective study, we observed that high MICs for vancomycin were associated with development of complicated MSSA CRBSI, regardless of the initial antimicrobial drug therapy used (vancomycin or β-lactams), which suggested that intrinsic characteristics of these strains might explain their pathogenic role in development of complicated bacteremia (*13*). We designed this multicenter prospective study to confirm the prognostic role of high MICs for vancomycin and to explore if increased MICs for other antistaphylococcal agents could also influence the risk for developing complicated MSSA CRBSI.

## Materials and Methods

### Study Design and Setting

We conducted a prospective, observational, multicenter study during April 2011−June 2014 in 5 hospitals in Spain in which we included patients given a diagnosis of MSSA CRBSI according to standard a definition (*14*) that fulfilled 5 criteria: 1) age >18 years and a life expectancy >7 days; 2) no use of daptomycin within the 3 months preceding the incident MSSA CRBSI episode; 3) removal of the intravascular catheter suspected to be the source of CRBSI within a maximum of 72 hours from sampling of the first blood cultures yielding MSSA; 4) a maximum of 72 hours of treatment with glycopeptides; and 5) at least the first blood culture isolate available for microbiological analysis. All included patients signed a study-specific informed consent form. The study was approved by the ethics research boards at all participating centers.

### Management of MSSA CRBSI

Although the nature of the study was not interventional, the adherence to evidence-based quality-of-care measures was strongly encouraged among participating investigators to optimize the therapeutic management of MSSA CRBSI. These measures were extraction of follow-up blood cultures, ordering echocardiography or venous Doppler ultrasound examination for patients with clinical indication, early use of intravenous cloxacillin or other highly effective antimicrobial drugs for treatment of for MSSA as definitive therapy, and adjustment of treatment duration according to the complexity of the infection.

### Definitions

Recorded clinical variables were age; site of acquisition of infection; concurrent conditions, as assessed by the Comorbidity Index of Charlson et al. (*15*); prognosis of the underlying disease (classified according to the McCabe and Jackson modified criteria as rapidly fatal [when death was expected within 3 months], ultimately fatal [when death was expected within a period of >3 months but <5 years] and nonfatal [when life expectancy was >5 years]) (*16*); type of intravenous catheter; timing of catheter removal; treatment administered; duration of fever; duration of bacteremia; Pitt bacteremia score (*17*); development of septic shock; endovascular infection or complicated bacteremia (defined below); and all-cause and MSSA CRBSI−attributable mortality rates at 30 days. MSSA CRBSI−attributable deaths at 30 days were identified as per the clinical judgment of the investigator.

Antimicrobial drugs were classified within 1 of the following mutually excluding categories: glycopeptides; daptomycin; antistaphylococcal β-lactams (parenteral cloxacillin, cefazolin or other active β-lactams [including parenteral amoxicillin/clavulanate, piperacillin/tazobactam, imipenem, or meropenem]); non−β-lactam agents with in vitro activity against MSSA (antistaphylococcal quinolones, tigecycline, or linezolid); and noneffective agents. Timing to initiation of antimicrobial drug therapy and duration of treatment were also recorded. Empiric therapy was defined as therapy administered before identification and antimicrobial susceptibility testing of the isolate. Complicated bacteremia (study outcome) was defined by 1 of 5 events occurring after the incident episode of MSSA CRBSI: 1) development of endocarditis (defined according to modified Duke criteria) (*18*); 2) septic thrombophlebitis (defined by persistent MSSA bacteremia for >72 hours after the initiation of active therapy associated to the documentation of a thrombus at the site of catheter insertion); 3) septic metastatic infection, including arthritis, spondylitis, infections involving vascular or osteoarticular prostheses (excluding intravascular catheter) or end-organ hematogenous seeding to other locations remote from the primary focus; 4) persistent bacteremia for >72 hours; or 5) persistent fever for >4 days after extraction of the first blood culture yielding MSSA, provided that alternative causes had been reasonably excluded (*19*).

### Microbiological Methods

Blood cultures were processed and isolates were identified according to standard techniques at each participating center. The first MSSA isolate from each patient was stored at −80°C until analysis and sent to the central reference laboratory (Department of Microbiology, University Hospital 12 de Octubre, Madrid, Spain). We subsequently subcultured strains twice and performed antimicrobial susceptibility testing by using the E-test method according to manufacturer’s recommendations (AB bioMérieux, Solna, Sweden). We tested vancomycin, daptomycin, oxacillin, and linezolid. In addition, the E-test macromethod was performed to screen for heteroresistant vancomycin-intermediate *S. aureus* (hVISA) (*20*). All the plates were visually read throughout the entire study period by 2 independent observers, who were blinded to each other and to the clinical outcomes. Interobserver discrepancies were resolved by reevaluation and a consensus decision.

### Statistical Analysis

We used the Student unpaired *t*-test to compare normally distributed continuous variables, the Mann-Whitney U test to compare continuous variables with non-normal distribution, and the χ^2^ and Fisher exact tests to compare proportions as appropriate. Areas under receiving operating characteristic curves for MICs for selected antistaphylococcal agents (on the basis of their discriminative value at the univariate comparisons) were plotted to select the optimal cutoff value in terms of sensitivity (S) and specificity (Sp) to distinguish between patients with or without complicated bacteremia by means of the Youden’s J statistic (J = S + Sp −1). We calculated the Pearson correlation coefficient (ρ) to assess the strength of the linear relationship among E-test MICs for daptomycin and vancomycin.

To test the effect of high MICs for antistaphylococcal agents and other clinical variables on the occurrence of complicated MSSA CRBSI, we plotted event-free Kaplan-Meier survival curves and performed comparisons between groups by using the log-rank test. Statistically relevant variables (p<0.10) at the univariate level were entered into multivariate Cox proportional hazard models with a backward stepwise selection, and hazard ratios (HRs) with 95% CIs were estimated. Some variables considered clinically relevant were also forced into the models regardless of their univariate p values. All statistical tests were 2-tailed, and the threshold of statistical significance was set at p<0.05. We used statistical software SPSS version 20.0 (SPSS, Inc., Chicago, IL, USA) to perform calculations of different analysis and generated graphics by using Prism version 6.0 (GraphPad Software Inc., La Jolla, CA, USA). Variables with an unacceptable proportion of missing values (>30%) were excluded from the analysis.

## Results

### Study Population and Outcome

Of 108 patients initially recruited, 19 were excluded because they did not fulfill inclusion criteria after revision of the clinical charts (13 had received >72 hours of treatment with vancomycin and 6 had had a delay of >3 days in catheter removal). An additional 6 patients cases were excluded because of inability to recover the MSSA strain for microbiological analysis. Therefore, we included 83 patients in the final analysis; median follow-up was 479 days (range 5 days–1,014 days). No variables were excluded because of missing data. None of the included patients had received vancomycin, daptomycin, oxacillin, or linezolid within the 3 months preceding the incidence MSSA CRBSI episode.

We compared the main demographic and clinical characteristics of the 83 patients in the final cohort (Table 1). Catheter removal was performed for all patients within the first 72 hours from sampling of the first blood cultures and, in most (75 [90.6%]) patients, within the first 48 hours. Antistaphylococcal β-lactams were used as empiric therapy in 43 patients (51.8%), glycopeptides in 32 (38.5%), and daptomycin in 17 (20.4%). Combined therapy was used for 25 (31.1%) patients. Twelve (14.5%) patients received no empiric therapy or the regimen administered was based on antimicrobial drugs with no in vitro activity against MSSA. For all patients who received daptomycin, the dose used was >6 mg/kg/day (range 6−10 mg/kg/day). Venous Doppler ultrasound examination and echocardiogram were performed for 41 (49.5%) and 61 patients (73.5%), respectively.

**Table 1:** Demographics and clinical characteristics of 83 patients with methicillin-sensitive *Staphylococcus aureus* catheter-related bloodstream infection*

Variable	Value
Age, y	60 ± 1.9
Male sex	49 (59.0)
Recruiting center	
1	32 (38.6)
2	19 (22.9)
3	2 (2.4)
4	12 (14.5)
5	18 (21.7)
Prognosis of underlying disease	
Not fatal	31 (37.3)
Fatal	44 (53.0)
Rapidly fatal	8 (9.6)
Charlson comorbidity index	3.7 ± 2.3
Previous conditions	
Diabetes	32 (38.6)
Malignancy	45 (54.2)
Valvular prosthesis	1 (1.2)
Ostheoarticular prosthesis	3 (3.6)
Renal failure requiring hemodialysis	10 (12)
Type of intravascular catheter	
Peripheral venous	32 (38.6)
Nontunneled (temporary) central venous	25 (30.1)
Peripherally inserted central	10 (12)
Permanent central venous	16 (19.3)
Pitt score at bacteremia onset	1.6 ± 1.4
Severe sepsis or septic shock	17 (20.5)
Empiric treatment including†	
Glycopeptides	32 (38.5)
Antistaphylococcal β-lactams‡	43 (51.8)
Other antistaphylococcal antimicrobial drugs	4 (4.8)
Daptomycin	17 (20.4)
None or noneffective antimicrobial drugs	12 (14.5)
Antimicrobial regimen	
Glycopeptides followed by antistaphylococcal β-lactam	25 (30.1)
Only antistaphylococcal β-lactams	30 (36.1)
Daptomycin followed by antistaphylococcal β-lactam	12 (14.5)
Only daptomycin	1 (1.2)
Glycopeptides followed by daptomycin plus antistaphylococcal β-lactam	7 (8.4)
Daptomycin plus antistaphylococcal β-lactam	7 (8.4)
Other	1 (1.2)
Timing of catheter removal	
Same day or before sampling first blood cultures	47 (56.6)
1 day after sampling	12 (14.5)
2 days after sampling	17 (20.5)
3 days after sampling	7 (8.4)
Venous Doppler ultrasound examination	41 (49.5)
Echocardiogram	61 (73.5)
Complicated MSSA CRBSI	26 (31.3)
Persistent fever >4 d§	11 (13.3)
Persistent bacteremia >72 h¶	6 (7.2)
Septic thrombophlebitis	10 (12)
Endocarditis	2 (2.4)
Hematogenous osteoarticular infection	2 (2.4)
Pulmonary emboli	2 (2.4)
All-cause deaths at 30 days	10 (12)
MSSA CRBSI−attributable deaths at 30 days	2 (2.4)

*Values are mean ± SD or no. (%). MSSA CRBSI: methicillin-sensitive *S. aureus* catheter-related bloodstream infection. †Some patients could be included in >1 category. ‡Parenteral cloxacillin, cefazolin, amoxicillin/clavulanate, piperacillin/tazobactam, imipenem, or meropenem. §Coincident with other forms of complicated bacteremia for 5 cases. ¶Coincident with other forms of complicated bacteremia in 5 cases.

Complicated MSSA CRBSI developed in 26 patients (31.3%) at a median of 4 days (interquartile range [IQR] 1–26 days) after sampling of blood cultures. No patients had late complications (i.e., >30 days). Septic thrombophlebitis was the most frequent complication (10 patients [12%]), followed by right-sided endocarditis (2 cases [2.4%]), hematogenous osteoarticular infection (2 cases [2.4%]), and pulmonary emboli (2 cases [2.4%]) (Technical Appendix Table 1). All-cause and MSSA CRBSI−attributable 30-day mortality rates were 12.0% and 2.4%, respectively.

### Correlation between MICs and Outcome

All strains were sensitive to all antimicrobial drugs tested. None of the isolates had the hVISA phenotype. The 50% MIC and 90% MIC and distribution ranges values were 0.5 μg/mL and 0.75 μg/mL (range 0.5–0.75 μg/mL) for cloxacillin, 1.5 μg/mL and 2 μg/mL (range 0.5–2.5 μg/mL) for vancomycin, 0.38 μg/mL and 0.75 μg/mL (range 0.125–0.94 μg/mL) for daptomycin, and 1 μg/mL and 1.5 μg/mL (range 0.25–2 μg/mL) for linezolid.

We compiled a comparative description of geometric means of MICs determined by E-test for vancomycin, daptomycin, oxacillin, and linezolid among isolates from patients with or without complicated bacteremia (Table 2). We observed that the MICs for vancomycin and daptomycin were higher for isolates from patients in whom complicated bacteremia developed.

**Table 2:** Antimicrobial susceptibility testing (E-test) of isolates from patients with methicillin-sensitive *Staphylococcus aureus* catheter-related bloodstream infection with or without complicated bacteremia*

Variable	Complicated bacteremia	
	No, n = 57	Yes, n = 26
MIC, μg/mL		
Vancomycin	1.2 ± 0.4†	1.5 ± 0.48†
Oxacillin	0.49 ± 0.27	0.51 ± 0.2
Daptomycin	0.4 ± 0.16†	0.5 ± 0.2†
Linezolid	1.08 ± 0.13	1.05 ± 0.08
Frequency		
Vancomycin MIC >1.5 μg/mL	25 (43.9)†	18 (69.2)†
Daptomycin MIC >0.5 μg/mL	4 (7.0)†	9 (34.6)†
Vancomycin MIC >1.5 μg/mL or daptomycin MIC >0.5 μg/mL	26 (45.6)†	19 (73.1)†
Vancomycin MIC >1.5 μg/mL and daptomycin MIC >0.5 μg/mL	3 (5.3)†	8 (30.8)†

*Values are geometric mean ± SD or no. (%). †p<0.05.

We subsequently performed an exploratory area under receiving operating characteristic curve analysis to establish the optimal cutoff point for the daptomycin MIC with the best discriminatory capacity to predict development of complicated bacteremia. An MIC for daptomycin of 0.5 μg/mL was selected (Technical Appendix Figure 1). This approach also confirmed that 1.5 μg/mL was the optimal cutoff point for the vancomycin MIC (Technical Appendix Figure 2). A total of 43 strains (41.8%) had a vancomycin MIC >1.5, μg/mL and 13 (15.7%) had a daptomycin MIC >0.5 μg/mL. On the basis of these thresholds, 11 (84.6%) of 13 isolates with high MICs for daptomycin also showed high MICs for vancomycin, and we found a moderate positive correlation between these variables (ρ = 0.21, p = 0.05) (Technical Appendix Figure 3). The percentage of MSSA strains with high MICs for vancomycin or daptomycin was higher in the group of patients with complicated bacteremia (Table 2). Strains isolated from all the patients in whom septic thrombophlebitis developed had high MICs for vancomycin or daptomycin.

We compared clinical characteristics of patients according to MICs for vancomycin or daptomycin (Table 3). Rates of complicated MSSA CRBSI were significantly higher among patients with episodes caused by strains with MICs for vancomycin (42% vs. 20%; p = 0.05) or daptomycin (69.2% vs. 24.3%; p = 0.004) than for those with episodes caused by strains with lower MICs. Strains with high MICs for vancomycin were not associated with higher rates of severe sepsis or septic shock at bacteremia onset, or with increased all-cause or attributable mortality rates. Conversely, we found that high MICs for daptomycin were more common among hemodynamically unstable episodes (i.e., severe sepsis or septic shock) than among remaining episodes (46.2% vs. 15.7%, p = 0.02). However, no effect on mortality rates was observed.

**Table 3:** Demographic and clinical characteristics of patients with methicillin-sensitive *Staphylococcus aureus* catheter-related bloodstream infection by MICs for vancomycin and daptomycin measured by E-test*

Variable	Vancomycin MIC, μg/mL				Daptomycin MIC, μg/mL		
	<1.5, n = 40	>1.5, n = 43	p value		<0.5, n = 70	>0.5, n = 13	p value
Age, y	61.9 ± 16.0	59.9 ± 19.0	0.42		60.2 ± 17.3	63.8 ± 21.0	0.5
Male sex	23 (57.5)	26 (60.5)	0.42		41 (58.6)	8 (61.5)	0.4
Prognosis of underlying disease							
Not fatal	18 (45.0)	13 (30.2)	0.3		24 (34.3)	7 (53.8)	0.3
Fatal	17(42.5)	27 (62.8)	0.1		39 (55.7)	5 (38.5)	0.4
Rapidly fatal	5 (12.5)	3 (6.9)	0.6		7 (10.0)	1 (7.7)	0.8
Charlson comorbidity index	3.5 ± 2.6	4.0 ± 2.1	0.31		3.7 ± 2.3	3.5 ± 2.1	0.9
Previous conditions							
Diabetes	20 (50.0)	12 (27.9)	0.06		28 (40)	4 (30.8)	0.6
Malignancy	17 (42.5)	28 (65.1)	0.12		37 (52.9)	8 (61.5)	0.5
Valvular prosthesis	0 (0.0)	1 (2.3)	0.9		1 (1.4)	0 (0.0)	0.9
Ostheoarticular prosthesis	1 (2.5)	2 (4.7)	0.79		2 (2.9)	1 (7.7)	0.6
Renal failure requiring hemodialysis	7 (17.5)	3 (6.9)	0.2		9 (12.9)	1 (7.7)	0.5
Type of intravascular catheter							
Peripheral venous	17 (42.5)	15 (34.9)	0.71		28 (40)	4 (30.8)	0.7
Nontunneled (temporary) central venous	13 (32.5)	12 (27.9)	0.82		19 (27.1)	6 (46.2)	0.3
Peripherally inserted central	5 (12.5)	5 (11.6)	0.76		10 (14.3)	0 (0.0)	0.3
Permanent central venous	5 (12.5)	11 (25.6)	0.2		13 (18.6)	3 (23.1)	0.6
Pitt score at bacteremia onset	1.5 ± 1.7	1.1 ± 1.4	0.9		1.3 ± 1.7	1.2 ± 1.0	0.9
Severe sepsis or septic shock	11 (27.5)	6 (13.9)	0.2		11 (15.7)	6 (46.2)	0.02
Empiric treatment including†							
Glycopeptides	16 (40.0)	16 (37.2)	0.7		27 (38.5)	5 (38.4)	0.7
Antistaphylococcal β-lactams‡	21 (52.5)	22 (51.1)	0.9		37 (52.8)	6 (46.1)	0.7
Other antistaphylococcal antimicrobial drugs	1 (2.5)	3 (6.9)	0.7		4 (5.7)	0 (0.0)	0.8
Daptomycin	7 (17.5)	10 (23.2)	0.8		13 (18.5)	4 (30.7)	0.5
None or noneffective antimicrobial drugs	7 (17.5)	5 (11.6)	0.8		10 (14.3)	2 (15.4)	0.8
Antimicrobial regimen							
Glycopeptides followed by antistaphylococcal β-lactam	11 (27.5)	14 (32.6)	0.7		21 (30.0)	4 (30.8)	0.9
Only antistaphylococcal β-lactams	14 (35)	16 (37.2)	0.8		26 (37.1)	4 (30.8)	0.8
Daptomycin followed by antistaphylococcal β-lactam	3 (7.5)	9 (20.9)	0.16		8 (11.4)	4 (30.8)	0.2
Only daptomycin	1 (2.5)	0 (0.0)	0.9		1 (1.5)	0 (0.0)	0.9
Glycopeptides followed by daptomycin plus antistaphylococcal β-lactam	6 (15.0)	1 (2.3)	0.09		6 (8.6)	1 (7.7)	0.9
Daptomycin plus antistaphylococcal β-lactam	4 (10.0)	3 (6.9)	0.8		7 (10)	0 (0.0)	0.5
Other	1 (1.25)	0 (0.0)	0.6		1 (2.5)	0 (0.0)	0.7
Timing of catheter removal							
Same day or before sampling first blood cultures	28 (70.0)	19 (44.2)	0.03		41 (58.6)	6 (46.2)	0.7
1 day after sampling	6 (15.0)	6 (13.9)	0.9		10 (14.3)	2 (15.4)	0.9
2 days after sampling	5 (12.5)	12 (27.9)	0.1		12 (17.1)	5 (38.5)	0.2
3 days after sampling	1 (2.5)	6 (13.9)	0.1		7 (10.0)	0 (0.0)	0.6
Complicated MSSA CRBSI	8 (20.0)	18 (41.9)	0.05		17 (24.3)	9 (69.2)	0.004
Persistent fever for >4 d	3 (7.5)	8 (18.6)	0.2		9 (12.9)	2 (15.4)	0.8
Persistent bacteremia for >72 h	2 (5.0)	4 (9.3)	0.7		5 (7.1)	1 (7.7)	0.9
Septic thrombophlebitis	1 (2.5)	9 (20.9)	0.02		4 (5.7)	6 (46.2)	<0.0001
Endocarditis	1 (2.5)	1 (2.3)	0.9		2 (2.9)	0 (0.0)	0.9
Hematogenous osteoarticular infection	1 (2.5)	1 (2.3)	0.9		1 (1.4)	1 (7.7)	0.5
Pulmonary emboli	2 (4.7)	0 (0.0)	0.3		2 (2.9)	0 (0.0)	0.6
All-cause deaths at 30 days	6 (15.0)	4 (9.3)	0.5		9 (12.9)	1 (7.7)	0.5
MSSA CRBSI–attributable deaths at 30 days	1 (2.5)	1 (2.3)	0.4		2 (2.9)	0 (0.0)	0.4

*Values are geometric mean ± SD or no. (%). MSSA CRBSI: methicillin-sensitive *Staphylococcus aureus* catheter-related bloodstream infection. †Some patients could be included in >1 category. ‡Parenteral cloxacillin, cefazolin, amoxicillin/clavulanate, piperacillin/tazobactam, imipenem, or meropenem.

### Risk Factors for Development of Complicated Bacteremia

We compared clinical characteristics of patients with or without complicated bacteremia (Table 4). Most (65.4%) patients with complicated cases were given a diagnosis at 1 recruiting center. Such an imbalance might be partially explained by the fact that such a center was the study promoter and the largest center at which the greatest diagnostic efforts were made. The probability of remaining free of complicated bacteremia at day 30 was significantly lower for patients with MSSA CRBSI caused by isolates with daptomycin MICs >0.5 μg/mL (33. 3% vs. 75%; log-rank test p = 0.002) and isolates with vancomycin MICs >1.5 μg/mL (59.2% vs. 79.6%; log-rank test p = 0.02) (Figure).

**Table 4:** Comparative analysis of patients with methicillin-sensitive *Staphylococcus aureus* catheter-related bloodstream infection with or without complicated bacteremia*

Variable	Complicated bacteremia		p value
	No, n = 5	Yes, n = 26	
Age, y	61.4 ± 16.0	59.3 ± 21.0	0.1
Male sex	32 (56.1)	17 (65.4)	0.5
Recruiting center			
1	15 (26.3)	17 (65.4)	0.01
2	14 (24.6)	5 (19.2)	0.8
3	2 (3.5)	0 (0.0)	1.0
4	11 (19.3)	1 (3.8)	0.2
5	15 (26.3)	3 (11.5)	0.2
Prognosis of underlying disease			
Not fatal	24 (42.1)	7 (26.9)	0.2
Fatal	29 (50.9)	15 (57.7)	0.6
Rapidly fatal	4 (7.0)	4 (15.4)	0.3
Charlson comorbidity index	3.6 ± 2.4	1.9 ± 1.9	0.3
Previous conditions			
Diabetes	20 (35.1)	12 (46.2)	0.3
Malignancy	29 (50.9)	16 (61.5)	0.6
Valvular prosthesis	1 (1.8)	0 (0.0)	1.0
Ostheoarticular prosthesis	1 (1.8)	2 (7.7)	0.2
Renal failure requiring hemodialysis	7 (12.3)	3 (11.5)	1.0
Type of intravascular catheter			
Peripheral venous	23 (40.4)	9 (34.6)	0.8
Nontunneled (temporary) central venous	17 (29.8)	8 (30.8)	1.0
Peripherally inserted central	7 (12.3)	3 (11.5)	1.0
Permanent central venous	10 (17.5)	6 (23.1)	0.6
Pitt score at bacteremia onset	1.1 ± 1.4	1.7 ± 1.9	0.1
Severe sepsis or septic shock	11 (19.3)	6 (23.1)	0.3
Empiric treatment including†			
Glycopeptides	24 (42)	8 (30.7)	0.4
Antistaphylococcal β-lactams‡	31 (54.3)	12 (46.1)	0.6
Other antistaphylococcal antimicrobial drugs	1 (1.7)	3 (11.5)	0.2
Daptomycin	8 (14)	9 (34.6)	0.06
Daptomycin monotherapy	5 (8.8)	8 (30.8)	0.02
None or noneffective antimicrobial drugs	10 (17.5)	2 (7.7)	0.4
Antimicrobial regimen			
Glycopeptides followed by antistaphylococcal β-lactam	19 (33.3)	6 (23.1)	0.4
Only antistaphylococcal β-lactams	23 (40.4)	7 (26.9)	0.3
Daptomycin followed by antistaphylococcal β-lactam	4 (7.0)	8 (30.8)	0.01
Only daptomycin	1 (1.8)	0 (0.0)	1.0
Glycopeptides followed by daptomycin plus antistaphylococcal β-lactam	6 (10.5)	1 (3.8)	0.4
Daptomycin plus antistaphylococcal β-lactam	3 (5.3)	4 (15.4)	0.2
Other	1 (1.8)	0 (0.0)	1.0
Timing of catheter removal			
Same day or before of sampling first blood cultures	36 (63.2)	11 (42.3)	0.1
1 day after sampling	6 (10.5)	6 (23.1)	0.1
2 days after sampling	10 (17.5)	7 (26.9)	0.4
3 days after sampling	5 (8.8)	2 (7.7)	1.0
Venous Doppler ultrasound examination	21 (36.8)	20 (76.9)	0.001
Echocardiogram	36 (63.2)	25 (96.2)	0.01
All-cause deaths at 30 days	7 (12.3)	3 (11.5)	1.0
MSSA CRBSI–attributable deaths at 30 days	0 (0.0)	2 (7.7)	0.09

*Values are geometric mean ± SD or no. (%). MSSA CRBSI: methicillin-sensitive *Staphylococcus aureus* catheter-related bloodstream infection. †Some patients could be included in >1 category. ‡Parenteral cloxacillin, cefazolin, amoxicillin/clavulanate, piperacillin/tazobactam, imipenem, or meropenem.

**Figure:**
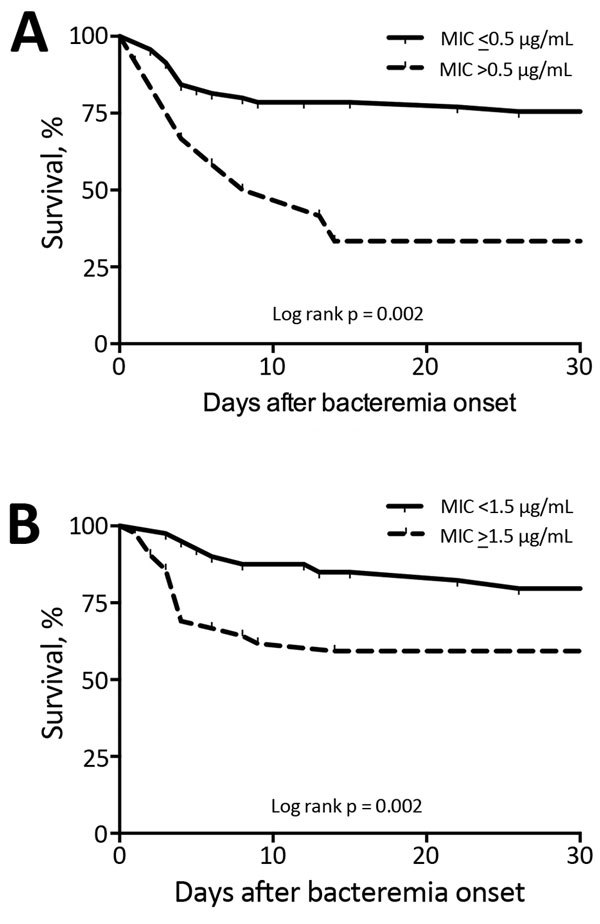
Event-free Kaplan-Meier survival curves for complicated bacteremia according to E-test MICs for daptomycin (A) and vancomycin (B) for complicated catheter-related bloodstream infections with a methicillin-sensitive *Staphylococcus aureus *isolate.

Because of low number of patients with complicated bacteremia (26 episodes), we could not assess the potential effect of MICs by performing a single logistic regression model adjusted for all the covariates found to be significant by univariate analysis. We alternatively attempted an exploratory approach based on different models. We used Cox regression models that incorporated a maximum of 3 variables at a time, always including the recruiting center as a potential confounder (Table 5; Technical Appendix Table 2). The presence of high MICs for vancomycin (minimum adjusted HR 2.4, 95% CI 1.2–5.5) and daptomycin (minimum adjusted HR 2.4, 95% CI 1.1–5.9) were kept as independent risk factors for development of complicated MSSA CRBSI in all models.

**Table 5:** Univariate and multivariate analyses of risk factors for development of complicated methicillin-sensitive *Staphylococcus aureus* catheter-related bloodstream infection*

Variable	Univariate, HR (95% CI)	Multivariate, HR (95% CI)†
Recruiting center 1	3.6 (1.6–8.1)	–
Any daptomycin-containing empiric therapy	2.5 (1.1–5.7)	–
Daptomycin monotherapy as empiric therapy	3.1 (1.3–7.1)	–
Vancomycin MIC >1.5 μg/mL	2.6 (1.1–5.9)	2.4 (1.2–5.5)
Daptomycin MIC >0.5 μg/mL	3.4 (1.6–8.2)	2.4 (1.1–5.9)

*HR, hazard ratio; –, corresponding variables were not retained in the final model. †Various models, including a maximum of 3 variables, were explored that combined all statistically significant variables identified at the univariate level. Only variables that were constantly retained in these models are shown with minimum HR values obtained.

We performed a sensitivity analysis by restricting the study outcome to the occurrence of septic thrombophlebitis. These associations remained essentially unchanged, and we observed even higher HRs (minimum adjusted HR for vancomycin MIC 6.0 [95% CI 0.7–52.2]; minimum adjusted HR for daptomycin MIC 5.5 [95% CI 1.3–22.5]) (Technical Appendix Tables 2, 3).

## Discussion

The aim of our study was to investigate the potential role of high MICs for vancomycin and other antistaphylococcal agents in predicting the risk for complications in a selected cohort of patients with MSSA CRBSI. We investigated this role after controlling for other well-defined prognostic factors, such as timing of catheter removal or appropriateness of empiric therapy.

We found a correlation between high MICs for vancomycin (>1.5 μg/mL) or daptomycin (>0.5 μg/mL) and an increased incidence of complicated bacteremia, particularly in terms of local endovascular complications represented by septic thrombophlebitis. Venous Doppler ultrasound examination was performed for only 50% of patients. Notwithstanding this potential drawback, we included in the definition of the study outcome the presence of persistent bacteremia in the absence of proven hematogenous spread to distant locations. This criterion might act as an appropriate clinical surrogate for identifying potential cases of septic thrombophlebitis that could have eventually remained undiagnosed.

Results confirm those obtained in our previous single-center retrospective study (*13*), in which we demonstrated that MSSA strains with vancomycin MICs >1.5 μg/mL were associated with a >2-fold increase in risk for development of complicated bacteremia and, specifically, a >6-fold increase in the risk for development of septic thrombophlebitis. In both studies, and in contrast to other authors who evaluated MSSA bacteremia from different sources (*7*), including patients with endocarditis (*8*), we did not find any apparent effect of increased MICs on sepsis severity or risk for death. The relatively low 30-day mortality rate for our selected cohort could partially account for this discrepancy. However, López-Cortés et al. (*12*) did not demonstrate any apparent effect of MICs for vancomycin on mortality rates in a recent study of a nonselected cohort of patients with MSSA bacteremia. These authors also reported an increased incidence of septic thrombophlebitis among patients with MSSA bacteremia caused by strains with high MICs for vancomycin. This combined evidence suggests that the theoretically increased pathogenicity of these strains might exert a local vascular effect but does necessarily imply a higher risk for severe sepsis or death.

The role of MICs for daptomycin as a prognostic marker in episodes of MSSA bacteremia has not been extensively investigated. In a study by Cervera et al., which included only patients with endocarditis caused by MSSA, MICs for daptomycin were not related to increased mortality rates (*8*). We have established a cutoff value for the MIC for daptomycin (>0.5 μg/mL) that also identified patients with an increased risk for development of complicated MSSA CRBSI, particularly septic thrombophlebitis. In accordance with lack of a correlation for the MIC for vancomycin, mortality rates did not differ according to MICs for daptomycin. Only 11 isolates (13.2% of the cohort) had simultaneously high MICs for vancomycin and daptomycin, and multivariate analysis demonstrated that both variables had an independent effect on the risk for development of complicated MSSA CRBSI.

It has been reported that MRSA isolates, mostly hVISA isolates, with higher MICs for vancomycin also have increased MICs for daptomycin (*21*–*24*). However, other studies have failed to confirm such an association (*25*–*27*). Pillai et al. found that some MSSA strains obtained from a patient with persistent bacteremia treated with vancomycin showed increasing MICs for daptomycin (*28*). In vitro studies suggested that patients infected with MSSA strains that have high MICs for vancomycin could have poor responses not only to vancomycin (*29*) but also to other antistaphylococcal agents, such as cloxacillin or daptomycin (*30*). In a recent in vitro study, daptomycin resistance was associated with increased thickness of bacterial cell wall peptidoglycan and a slightly delayed transition to the postexponential growth phase because of alterations in bacterial metabolism (*31*). These findings suggest a deleterious fitness cost in terms of invasiveness (*31*) but also a blockage in the adhesive phase of *S. aureus* that might be linked to an increased trend in local complications. High MICs for vancomycin in isolates with the hVISA phenotype have been shown to be associated with increased persistence, rather than increased mortality rates and sepsis in patients (*32*). Although we did not detect any hVISA isolates in our study, we postulate that a high MIC for vancomycin could be an essential step in acquisition of this phenotype, and that this step might be the common underlying pathogenic mechanism.

Apart from the limited number of events (which prevented including all explanatory variables into a single multivariate model), some limitations of the study also deserve specific consideration. Despite its multicenter nature, 1 specific participating center provided most of the results because this center recruited most of the patients and most patients who had complicated bacteremia. However, all multivariate models were adjusted by the recruiting center to minimize such potential bias. Antimicrobial drug susceptibility testing was not performed in parallel with the reference broth microdilution method, which could have enabled us to confirm the poor correlation previously reported between this technique and the E-test (*11*).

Conversely, interobserver reproducibility of the E-test for assessing vancomycin MICs has been recently questioned (*33*). In our study, MIC testing was performed over a continuous period, and E-test results were read by 2 independent observers who were blinded to patient outcome, an approach that theoretically would reduce intraassay variability and facilitate interpretation of results. Finally, real-life applicability of our findings and potential prognostic value of performing E-tests for daptomycin for all MSSA isolates from patients with CRBSI remain to be established with support of cost-effectiveness analysis. Although we did not specifically address some variables that had been postulated to be related with higher MICs for vancomycin or daptomycin, such as inoculum size (*34*) or use of suboptimal dosing of daptomycin for cases of persistent bacteremia (*35*), we set a priori inclusion criteria to homogenize the clinical management of patients analyzed (including uniform dosing of daptomycin and vancomycin).

In summary, our data suggest that patients with MSSA CRBSI caused by strains with MICs >1.5 μg/mL for vancomycin or >0.5 μg/mL for daptomycin determined by E-test are more prone to show development of complications, particularly septic thrombophlebitis. However, these patients do not appear to have an increased risk for severe sepsis or death. On the basis of these preliminary findings, patients with CRBSI cause by MSSA isolates with high MICs for vancomycin or daptomycin should be carefully evaluated to exclude local complications and eventually individualize duration of therapy. These prospects would merit confirmation in future intervention studies.

Technical Appendix Additional information for high MICs for vancomycin and daptomycin and complicated catheter-related bloodstream infections with methicillin-sensitive *Staphylococcus aureus*.
