# Supplementary material for: High MICs for Vancomycin and Daptomycin and Complicated Catheter-Related Bloodstream Infections with Methicillin-Sensitive Staphylococcus aureus
Source: Emerg Infect Dis. 2016 Jun;22(6):1057–66. doi: 10.3201/eid2206.151709 (PMC4880091; doi:10.3201/eid2206.151709)
Supplement: Technical Appendix — Additional information for high MICs for vancomycin and daptomycin and complicated catheter-related bloodstream infections with methicillin-sensitive Staphylococcus aureus. [file 15-1709-Techapp-s1.pdf]

Article DOI: http://dx.doi.org/10.3201/eid2206.15-1709

High MICs for Vancomycin and Daptomycin and Complicated Catheter-Related Bloodstream Infections with Methicillin-Sensitive *Staphylococcus aureus*

Technical Appendix

Technical Appendix Table 1. Comparative analysis of patients with methicillin-sensitive *Staphylococcus aureus* catheter-related bloodstream infection with or without septic thrombophlebitis*

	Septic thrombophlebitis		
Variable	No (n = 73)	Yes (n = 10)	p value
Age, y	61.0 ± 16.1	58.7 ± 18.3	0.3
Male sex	42 (57.5)	7 (70.0)	0.6
Recruiting center			
1	24 (32.9)	8 (80.0)	0.01
2	17 (23.3)	2 (20.0)	0.7
3	2 (2.7)	0 (0.0)	1.0
4	12 (16.4)	0 (0.0)	0.3
5	18 (24.7)	0 (0.0)	0.1
Prognosis of underlying disease			
Not fatal	28 (38.4)	3 (30.0)	0.7
Fatal	38 (52.1)	6 (60.0)	0.7
Rapidly fatal	7 (9.6)	1 (10.0)	1.0
Charlson comorbidity index	3.8 ± 2.3	3.4 ± 2.1	0.9
Previous conditions			
Diabetes	28 (38.4)	4 (40.0)	1.0
Malignancy	39 (53.4)	6 (60.0)	0.7
Valvular prosthesis	1 (1.4)	0 (0.0)	1.0
Ostheoarticular prosthesis	3 (4.1)	0 (0.0)	1.0
Renal failure requiring hemodyalisis	9 (12.3)	1 (10.0)	1.0
Type of intravascular catheter			
Peripheral venous	28 (38.4)	4 (40.0)	1.0
Nontunneled (temporary) central venous	23 (31.5)	2 (20.0)	0.7
Peripherally inserted central	8 (11)	2 (20.0)	0.3
Permanent central venous	14 (19.2)	2 (20.0)	1.0
Pitt score at bacteremia onset	1.3 ± 1.6	1.1 ± 1.2	0.2
Severe sepsis or septic shock	15 (20.5)	2 (20.0)	1.0
Empiric treatment including†			
Glycopeptides	27 (38.5)	5 (50.0)	0.4
Antistaphylococcal β-lactams‡	39 (55.7)	4 (40.0)	0.5
Other antistaphylococcal antimicrobial drugs	3 (4.3)	1 (10.0)	0.4
Daptomycin	14 (19.2)	3 (30.0)	0.4
None or noneffective antimicrobial drugs	11 (15.1)	1 (10.0)	1.0
Antimicrobial drug regimen			
Glycopeptides followed by antistaphylococcal β-lactam	21 (28.8)	4 (40)	0.5
Only antistaphylococcal β-lactams	27 (37)	3 (30.0)	0.7
Daptomycin followed by antistaphylococcal β-lactam	9 (12.3)	3 (30.0)	0.15
Only daptomycin	1 (1.4)	0 (0.0)	1.0
Glycopeptides followed by daptomycin plus antistaphylococcal β-lactam	7 (9.6)	0 (0.0)	0.6
Daptomycin plus antistaphylococcal β-lactam	7 (9.6)	0 (0.0)	0.6
Other	1 (1.4)	0 (0.0)	1.0
Timing of catheter removal			
Same day or before sampling first blood cultures	42 (57.5)	5 (50.0)	0.7
1 day after sampling	11 (15.1)	1 (10.0)	1.0
2 days after sampling	13 (17.8)	4 (40.0)	0.2
3 days after sampling	7 (9.6)	0 (0.0)	0.6
Severe sepsis or septic shock	11 (15.1)	6 (60.0)	0.004
Echocardiogram	51 (69.8)	10 (100.0)	0.06
All-cause deaths at 30 days	9 (12.3)	1 (10.0)	.0
MSSA CRBSI–attributable deaths at 30 days	2 (2.7)	0 (0.0)	1.0

Variable	Septic thrombophlebitis		p value
	No (n = 73)	Yes (n = 10)	

*Values are mean ± SD or no. (%). MSSA CRBSI: methicillin-susceptible *S. aureus* catheter-related bloodstream infection.
†Some patients could be included in >1 category.
‡Parenteral cloxacillin, cefazolin, amoxicillin/clavulanate, piperacillin/tazobactam, imipenem, or meropenem.

Technical Appendix Table 2. Univariate and multivariate analyses of risk factors for development of septic thrombophlebitis in patients with methicillin-sensitive *Staphylococcus aureus* catheter-related bloodstream infection*

Variable	Univariate, HR (95% CI)	Multivariate, HR (95% CI)†
Recruiting center 1	7.3 (1.5–34.0)	–
Any daptomycin-containing empiric therapy	2.5 (1.1–5.7)	–
Daptomycin monotherapy as empiric therapy	3.1 (1.3–7.1)	–
Vancomycin MIC ≥1.5 μg/mL	10.1 (1.3–91.0)	6.0 (0.7–52.2)
Daptomycin MIC >0.5 μg/mL	8.4 (2.4–29.1)	5.5 (1.3–22.5)

*HR, hazard ratio; –, corresponding variables were not retained in the final model.
†Various models, including a maximum of 3 variables, were explored that combined all statistically significant variables identified at the univariate level. Only variables that were constantly retained in these models are shown with minimum HR values obtained.

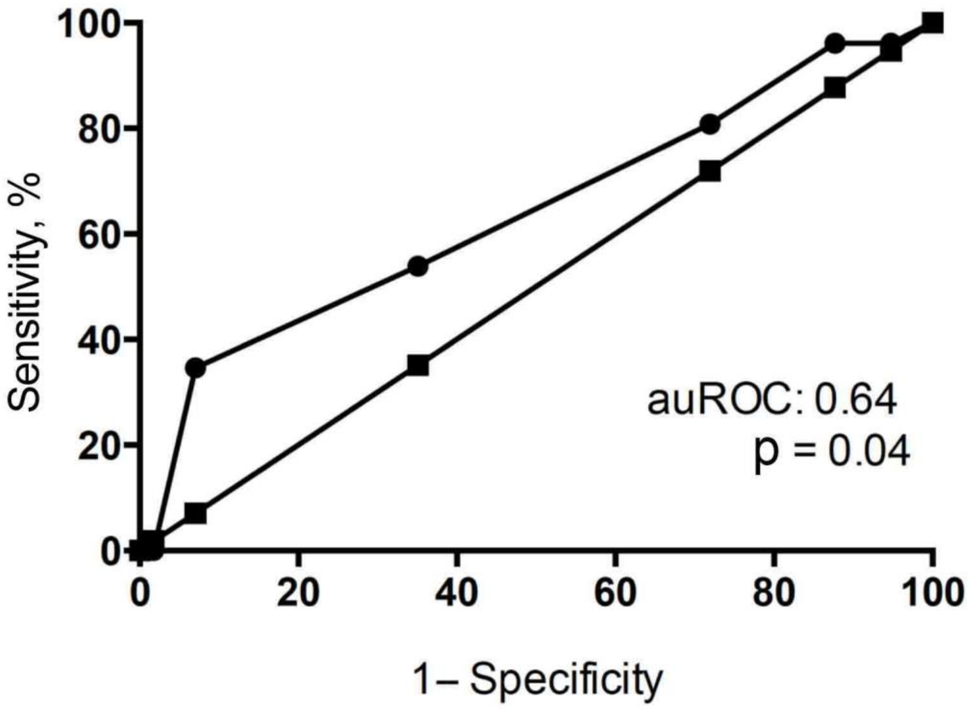

Technical Appendix Figure 1. Receiving operating characteristics curve of MICs (measured by E-test) for daptomycin in predicting development of complicated bacteremia in patients with methicillin-sensitive *Staphylococcus aureus* catheter-related bloodstream infection. auROC, area under receiving operating characteristic.

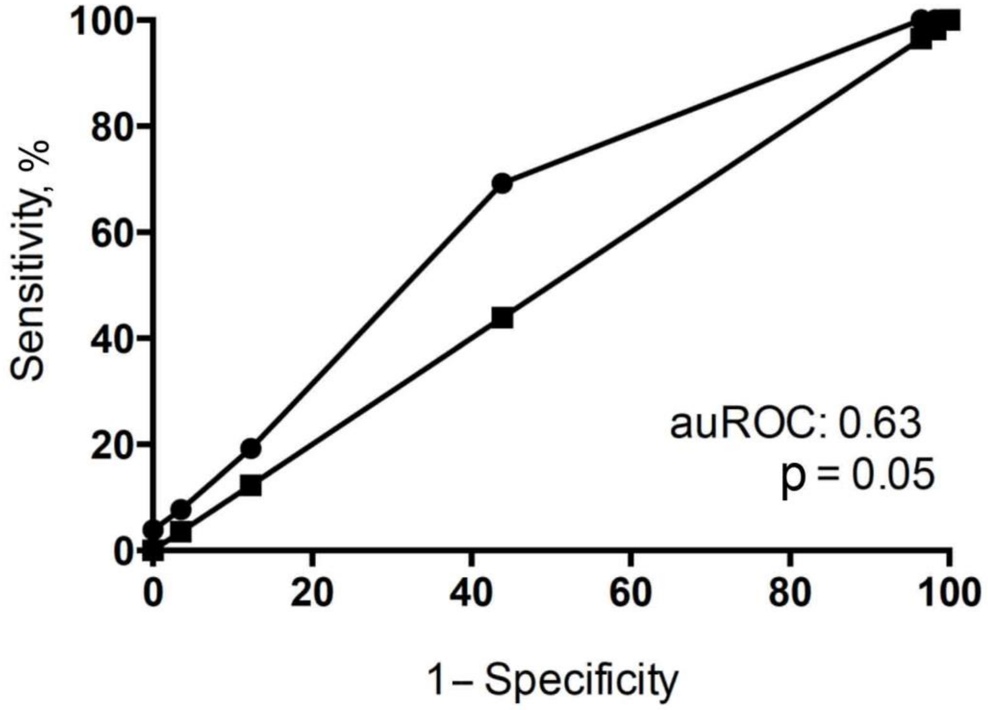

Technical Appendix Figure 2. Receiving operating characteristics curve of MICs (measured by E-test) for vancomycin in predicting development of complicated bacteremia in patients with methicillin-susceptible *Staphylococcus aureus* catheter-related blood stream infection. auROC, area under receiving operating characteristic.

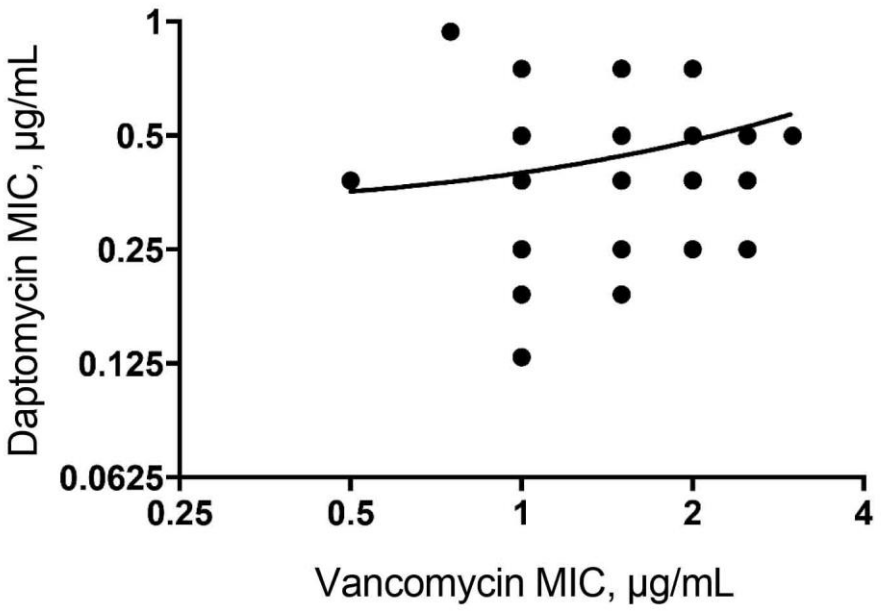

Technical Appendix Figure 3. Pearson correlation between MICs for vancomycin and daptomycin ($\rho = 0.21$, $p = 0.05$) in predicting development of complicated bacteremia in patients with methicillin-sensitive *Staphylococcus aureus* catheter-related bloodstream infection.
